# Evaluation and optimization of novel extraction algorithms for the automatic detection of atrial activations recorded within the pulmonary veins during atrial fibrillation

**DOI:** 10.1186/s12911-022-01969-5

**Published:** 2022-08-28

**Authors:** Yann Prudat, Adrian Luca, Sasan Yazdani, Nicolas Derval, Pierre Jaïs, Laurent Roten, Benjamin Berte, Etienne Pruvot, Jean-Marc Vesin, Patrizio Pascale

**Affiliations:** 1 Applied Signal Processing Group, Swiss Federal Institute of Technology, Lausanne, Switzerland; 2 Department of Cardiology, Lausanne University Hospital and University of Lausanne, Lausanne, Switzerland; 3 Hôpital Cardiologique du Haut-Lévêque and Université de Bordeaux, IHU LYRIC ANR-10-IAHU-04, Bordeaux-Pessac, France; 4 Department of Cardiology, Inselspital, Bern University Hospital, University of Bern, Bern, Switzerland; 5 Heart Center, Luzerner Kantonsspital, Lucerne, Switzerland

**Keywords:** Biomedical signal processing, Non-linear signal processing, Atrial fibrillation, Intracardiac electrograms, Activation detection

## Abstract

**Background and objective:**

The automated detection of atrial activations (AAs) recorded from intracardiac electrograms (IEGMs) during atrial fibrillation (AF) is challenging considering their various amplitudes, morphologies and cycle length. Activation time estimation is further complicated by the constant changes in the IEGM active zones in complex and/or fractionated signals. We propose a new method which provides reliable automatic extraction of intracardiac AAs recorded within the pulmonary veins during AF and an accurate estimation of their local activation times.

**Methods:**

First, two recently developed algorithms were evaluated and optimized on 118 recordings of pulmonary vein IEGM taken from 35 patients undergoing ablation of persistent AF. The adaptive mathematical morphology algorithm (AMM) uses an adaptive structuring element to extract AAs based on their morphological features. The relative-energy algorithm (Rel-En) uses short- and long-term energies to enhance and detect the AAs in the IEGM signals. Second, following the AA extraction, the signal amplitude was weighted using statistics of the AA sequences in order to reduce over- and undersensing of the algorithms. The detection capacity of our algorithms was compared with manually annotated activations and with two previously developed algorithms based on the Teager–Kaiser energy operator and the AF cycle length iteration, respectively. Finally, a method based on the barycenter was developed to reduce artificial variations in the activation annotations of complex IEGM signals.

**Results:**

The best detection was achieved using Rel-En, yielding a false negative rate of 0.76% and a false positive rate of only 0.12% (total error rate 0.88%) against expert annotation. The post-processing further reduced the total error rate of the Rel-En algorithm by 70% (yielding to a final total error rate of 0.28%).

**Conclusion:**

The proposed method shows reliable detection and robust temporal annotation of AAs recorded within pulmonary veins in AF. The method has low computational cost and high robustness for automatic detection of AAs, which makes it a suitable approach for online use in a procedural context.

## Background and objective

Atrial fibrillation (AF) is the most common arrhythmia in clinical practice. Morbidity and mortality associated with AF place a considerable burden on society related mostly to thromboembolic and hemodynamic complications. Catheter-based ablation has now evolved to become an important treatment option for many patients with AF. The analysis of intracardiac electrograms (IEGMs) during ablation procedures provides important information in order to identify potential ablation targets and predict ablation outcome. More specifically, characteristics such as the AF cycle length (CL) of atrial activations (AAs), and its dynamic patterns, have been shown to help identify critical ablation targets or anticipate ablation outcome [1–5]. Similarly, the recording of intermittent rapid atrial activities in the pulmonary veins (PVs) has been shown to identify arrhythmogenic foci that trigger AF [6, 7]. The analysis of PVs activity may also provide clues to better define ablation targets and ablation endpoint in chronic forms of AF [8, 9].

In order to perform analysis of the AF CL and of the patterns of AA times series, an effective automatic extraction algorithm is the first step needed. Various algorithms based on amplitude thresholds [10, 11], energy operators [12] or template matching [13] have been proposed, but automatic detection of individual activations is technically challenging when dealing with AF IEGMs exhibiting a large patient- and time-dependent spectrum of morphologies, amplitudes and frequencies.

Another limitation is the technical difficulty of correctly estimating the local activation times (LATs) of the detected AAs. The constant changes in the IEGM active zones observed in complex and/or fractionated signals will induce variations in the temporal analysis of AA sequences. The maximum and/or minimum peaks or the maximum slope have been traditionally used to estimate LATs [14, 15]. These methods may provide unreliable or biased estimates of the LAT sequence, especially in the presence of complex or fractionated signal morphologies, such as in AF recordings.

We recently developed two algorithms to extract biomedical events in real-time/online applications [16, 17]. The purpose of our study is therefore to (1) evaluate these two approaches in order to identify an algorithm able to provide reliable automatic extraction of intracardiac AAs recorded within the pulmonary veins during AF, and (2) propose a robust method to estimate the LATs of the detected AA sequences in the presence of complex and/or fractionated signals. We compared our methods with manually annotated activations and with two other previously developed algorithms based on the Teager-Kaiser energy operator [18] and the AF cycle length iteration [19], respectively.

## Methods

**Fig. 1:**
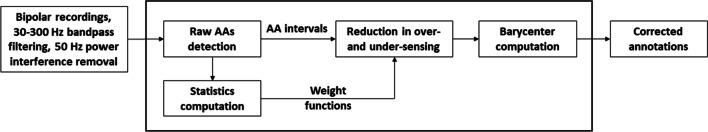
Block diagram of the detection method

To obtain the raw AA detections from IEGM, we applied two innovative algorithms: (1) a short-term event extraction algorithm named Relative-Energy (Rel-En) [16], and (2) a mathematical morphology approach with an adaptive structuring element called Adaptive Mathematical Morphology (AMM) [17]. Following the raw AA detection, we further used the statistics of the extracted AA intervals to reduce the over- and undersensing of AA detections. Then, we used a barycenter-based method to correctly estimate the LAT. Figure 1 illustrates the workflow used in the present study.

### Electrogram dataset

The electrogram dataset consisted of 118 intracardiac bipolar recordings collected in 35 patients (Age [IQR]: 61 [59–68] years old) undergoing first time ablation of persistent AF (sustained duration of AF 12 [7–24] months) in three different hospitals (CHUV, Lausanne, Switzerland; Inselspital, Bern, Switzerland; CHU de Bordeaux, Bordeaux, France). All patients provided written informed consent and the study was approved by the Local Human Research Ethics Committees.

The recordings were made using a 20-pole variable circumferential Lasso® catheter (Biosense Webster, Diamond Bar, CA) with closely spaced electrodes (2–6–2 mm) placed in the PVs prior to radiofrequency ablation. The data were obtained via the EP recording system (Labsystem Pro, Boston Scientific, Lowell, MA, for Bordeaux data; and Axiom Sensis XP®, Siemens, Berlin, Germany, for Lausanne and Bern data). The bipolar IEGMs were recorded during the electrophysiological protocol at a sampling rate of 1 or 2 kHz (band-pass filtered 30–300 Hz, 50 Hz notch filtered). The 118 IEGM segments had a mean duration of 43 ± 18 s (median 49 s; range 9–75 s).

In order to test the performance of the algorithms, a blinded clinical electrophysiology expert manually annotated the exact LATs on the recordings using an in-house Matlab-based graphical user interface. These manual annotations were used as the ground truth. The database was randomly distributed into a training cohort (22 patients, 78 recordings, total IEGM duration of 3525 s, total number of 28,136 AAs) to optimize the algorithm, and a validation cohort (13 patients, 40 recordings, total IEGM duration of 1527 s, total number of 19,090 AAs) to compute detection efficiency. The two cohorts were created patient-wise while aiming to keep a 2:1 ratio in the number of recordings (i.e. each cohort contains IEGM signals from different patients). Random patients (using built-in uniform random generator in Matlab) were assigned to the validation cohort until the number of recordings reached 1/3 of the total number. Clinical characteristics were similar between the two cohorts and are presented in Table 1. The significance of any difference between subgroups was analysed with the Mann‐Whitney U test for continuous variables, and with Fisher’s exact test for categorical variables.

**Table 1:** Clinical characteristics of the training and validation cohorts. Data are shown as median and interquartile range for continuous variable and counts for categorical variables

	All (n = 35)	Training (n = 22)	Validation (n = 13)	Training versus validation *p* value
No. of IEGM recordings	118	78	40	
Total number of AA	28,136	19,090	9046	
Total IEGM duration (s)	5052	3525	1527	
Duration of IEGM recordings (s)	42.8 [25.4–58.5]	45.2 [29.2–61.2]	38.2 [18.3–58.3]	0.19
Age (years)	61.0 [59.0–68.0]	61.5 [57.0–67.8]	61.0 [59.0–68.8]	0.79
Sex (male/female)	26/9	17/5	9/4	0.69
AF duration (years)	4.0 [1.8–6.5]	3.0 [1.1–5.8]	6.0 [4.0–10.0]	0.05
Duration of sustained AF (months)	12.0 [7.2–24.0]	12.0 [8.0–24.0]	12.0 [7.0–24.0]	0.77
LVEF (%)	55.0 [49.0–62.5]	57.5 [48.5–63.8]	55.0 [50.0–61.0]	0.95
Left atrial size (mm)	47.5 [40.8–52.3]	47.0 [43.0–49.0]	48.0 [40.0–53.0]	0.65

### Raw detection of atrial activations

#### Relative energy algorithm

**Fig. 2:**
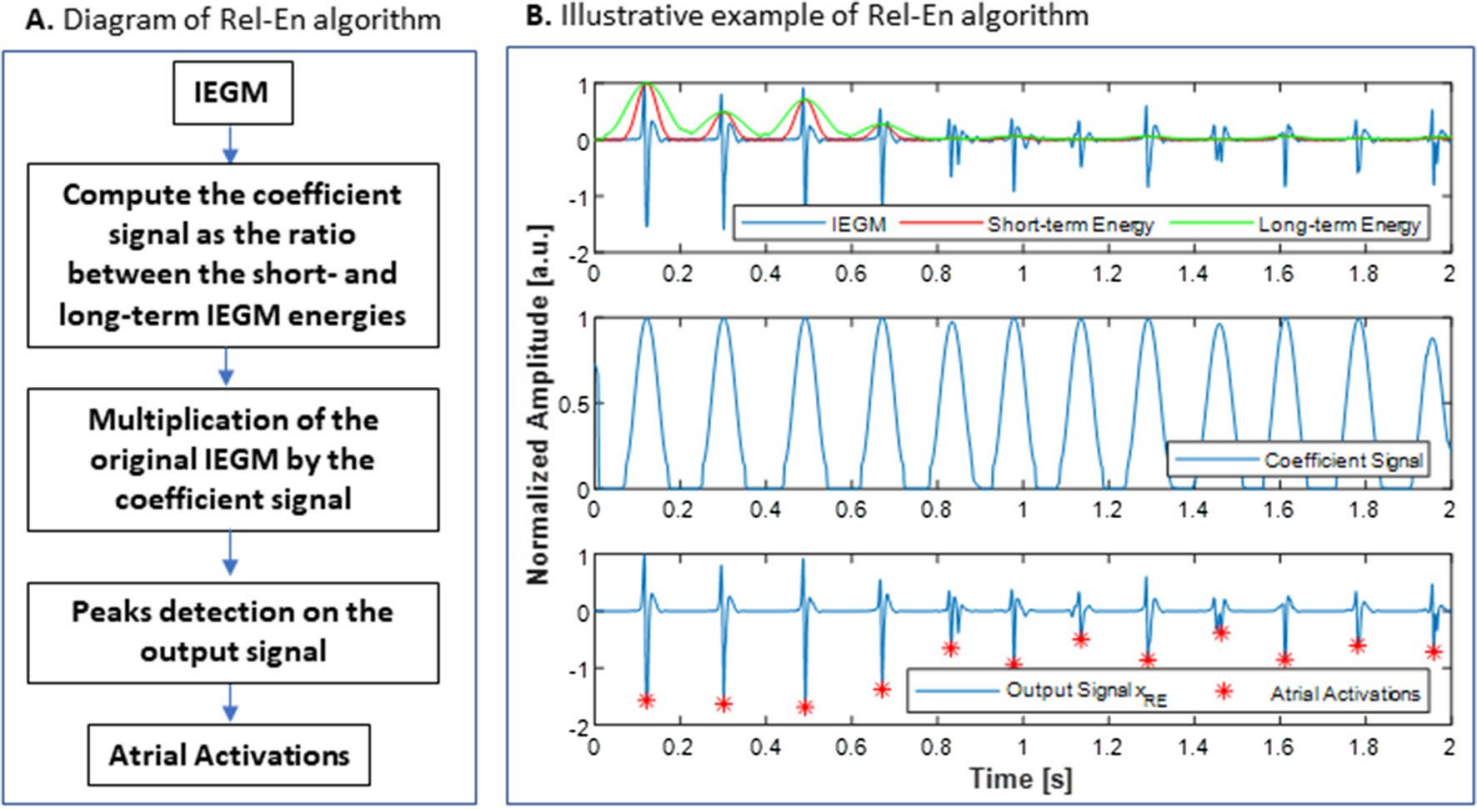
Block diagram (**A**) and illustrative example (**B**) of the relative energy algorithm

The relative-energy (Rel-En) algorithm is an original short-term event detection algorithm proposed recently [16]. In this approach, illustrated in Fig. 2, the input signal is enhanced by multiplication with a signal-derived coefficient to allow easier and more accurate detection of the AA. The enhancing coefficient signal $c\left( \cdot \right)$ is computed as the ratio between the short- and long-term energies of the input signal $x\left( \cdot \right)$:

1 $$c\left( n \right) = \frac{{\mathop \sum \nolimits_{{i = n - s_{win} }}^{{n + s_{win} }} \left| {x\left( i \right)} \right|^{p} }}{{\mathop \sum \nolimits_{{j = n - l_{win} }}^{{n + l_{win} }} \left| {Hamming\left( j \right) \cdot x\left( j \right)} \right|^{p} }}$$

where $s_{win}$ and $l_{win}$ represent the half-length of the short and long sliding windows, respectively. The windowing is performed using a Hamming window. While the short-term window duration allows for the extraction of the AA, the long-term window duration reflects the local baseline behavior of the electrogram. The parameter *p* denotes the exponent. Since the AAs can be intermixed with complex and/or fractionated signals, small values of *p* can lead to a high number of false positives. In contrast, a larger *p* has the tendency to improve the extraction of the AAs when higher levels of perturbation are present, but this may lead to missed detections of low amplitude AAs. In the present study the exponent parameter *p* was set to 4, as it was shown in [16] that the detection error rate does not drastically change around the optimal parameter. Finally, the output signal is computed as:

2 $$x_{RE} \left( n \right) = x\left( n \right) \cdot c\left( n \right)$$

AAs are detected on $x_{RE}$ signal using a minimum threshold $TH_{P}$ ad hoc, defined as the $P$-th percentile of the amplitude distribution of $x_{RE}$. The three parameters $s_{win}$, $l_{win}$ and $P$ are signal-dependent and need to be tuned to achieve efficient detection. To avoid the detection of multiple peaks during one activation, a minimum interval between two consecutive activations must be defined. A 70 ms interval was selected based on the shortest reported interval physiologically recorded in PVs [20].

#### Adaptive mathematical morphology algorithm

The adaptive mathematical morphology (AMM) algorithm has been thoroughly presented in previous papers aiming at either extraction of QRS complexes from the surface ECG [17] or detection of AA sequences from intracardiac signals [21]. Briefly, the AAs are extracted using a structuring element (SE) which is continuously updated for each new AA based on the topological features of the previous detected AA. The AMM algorithm consists of the following steps (Fig. 3A):

1. A synthesized SE with an AA-like morphology is empirically defined using the IEGM at hand (Fig. 3B). The SE is defined by five fiducial points representing the onset, offset, peak, the minimum between the onset and the peak, and the minimum between the peak and the offset. The amplitude of the synthesized SE is computed as the difference between the maximum and the minimum value of the first 500 ms of the IEGM. The duration of the synthesized SE is empirically chosen. In the present work, we will test a range of SE lengths to find the optimal duration for an efficient AA detection (Section III Results).
2. The IEGM is split in 200-ms non-overlapping sliding windows. Using the SE, the average of the mathematical morphology operators top-hat and bottom-hat is calculated on each 200-ms IEGM epoch (Eq. 3). The top-hat and bottom-hat operators are based on two basic morphological operation, dilation and erosion, and the combined operators opening and closing [22]. The first 200-ms IEGM window is filtered using the synthesized SE, afterwards the following windows are filtered using an updated SE (step 4). Figure 3C shows that each filtering phase results in a feature signal $x_{MM}$ which consists in non-zero values at the times of AA and zero otherwise.
  3 $$x_{MM} = x - \frac{x \circ SE + x \cdot SE}{2},$$

**Fig. 3:**
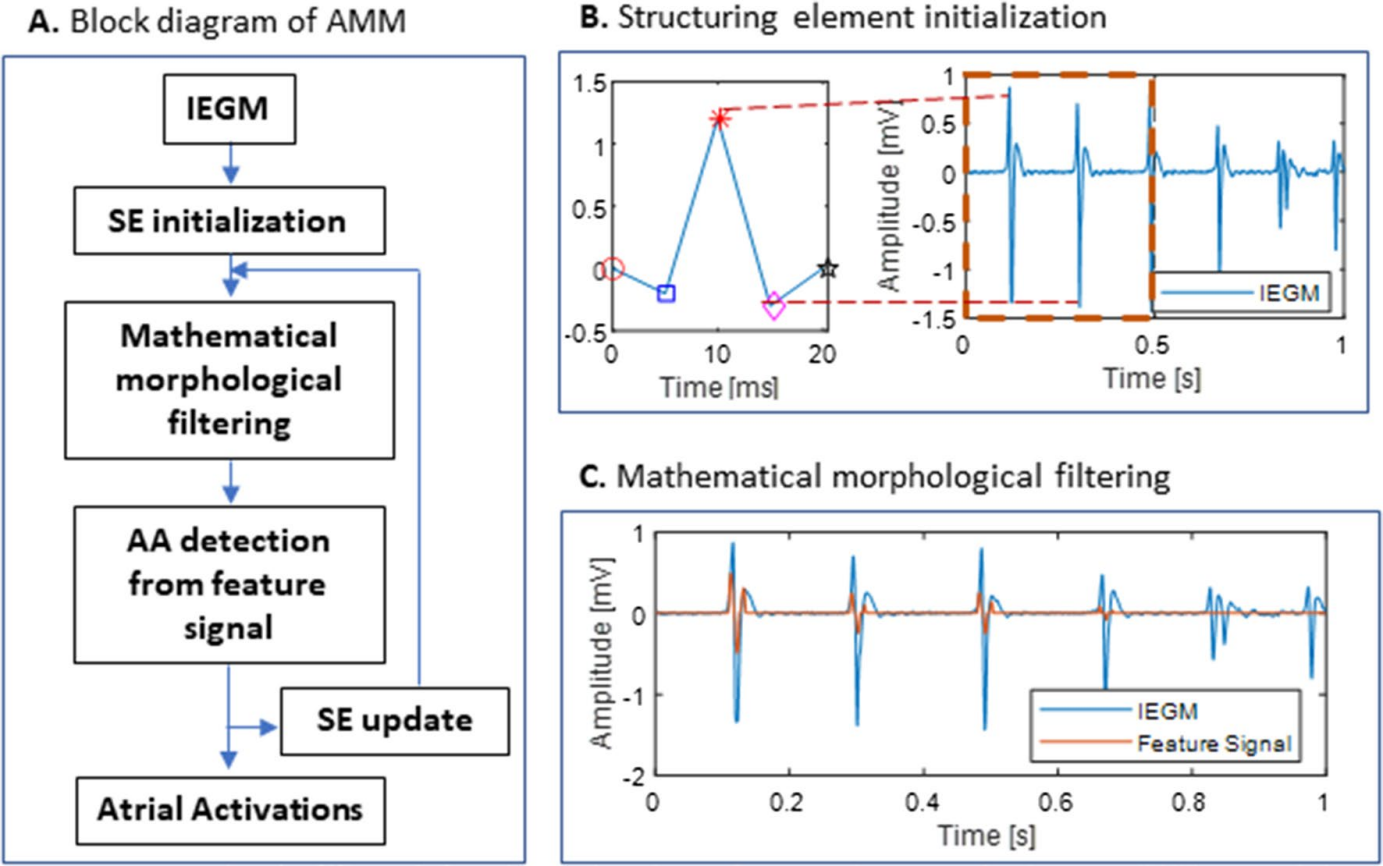
**A** Block diagram of the AMM algorithm. **B** Initialization of the SE. The fiducial points of the SE are illustrated in the left panel: ○—the onset, □—the minimum between the onset and the main peak, *—the main peak, ◇—the minimum between the main peak and the offset, □—the offset. **C** Mathematical morphological filtering. The feature signal represents the average of the mathematical morphology operators top-hat and bottom-hat calculated on each 200-ms IEGM epoch. AMM, adaptive mathematical morphology; IEGM, intracardiac electrogram, SE, structuring element
  where x represents the 200-ms IEGM to which the mathematical morphology filtering is applied, the symbols ∘ and ⦁ denote the opening and closing operation respectively.
3. Following the filtering phase of each window, the feature signal $x_{MM}$ is scrutinized to extract the AAs. The non-zero segments are identified and processed as follows:
  The most significant peak in the extracted segment is defined as the AA. To prevent false AA extraction, the distance between two successive detected AAs must be at least 70 ms (physiological limit).
  Onset and offset of the newly detected AA are considered as the start and end of the non-zero segment.
  The minimum between the onset and the significant peak, together with the minimum between the significant peak and the offset are extracted for the SE update (step 4).
4. The location indices and amplitude values of the five fiducial points (AA onset, offset, and peak, and the local minima around the onset and offset) extracted at step (3) are used to update the SE so that it best represents the actual AA morphology of the subject:
  4 $$\begin{aligned} NewLocation & = \left( {1 - \alpha } \right) \cdot CurrentLocation \\ & \quad + \alpha \cdot ExtractedLocation \\ NewAmplitude & = \left( {1 - \alpha } \right) \cdot CurrentAmplitude \\ & \quad + \alpha \cdot ExtractedAmplitude \\ \end{aligned}$$

*CurrentLocation* and *ExtractedLocation* represent the location indices of the first minimum, peak, second minimum and offset of the current SE and the extracted AA, respectively. The *CurrentLocation* is computed as the distance from the fiducial point to the onset of the current SE, and the *ExtractedLocation* is computed as the distance from the fiducial point to the onset of the extracted AA (the onset of the non-zero segment). *CurrentAmplitude* and *ExtractedAmplitude* represent the amplitude of the offset, first minimum, peak, second minimum and offset of the current SE and the extracted AA, respectively. Using *NewLocation* and *NewAmplitude*, SE is updated by means of linear interpolation. The newly updated SE is then used for filtering of the next IEGM window. An α value of 0.5 was used as learning coefficient to avoid excessive variations of SE, for instance in case of large AA amplitude changes.

### Reduction in over- and undersensing of atrial activations

Due to the various morphologies of the AAs in AF and the beat-to-beat variability in morphology and amplitude, a substantial amount of activations may be missed, or noise, artifacts or far-field components may be considered as activations. We therefore aimed to design a post-processing method able to reduce the oversensing, undersensing, and total error rate of the detection algorithms. To this end, the signal amplitude before and after a detected activation is weighted in order to spot a missed or false detection according to the probability that it represents a true activation. Two weight functions, one linear ($w_{l}$) and one non-linear ($w_{n}$), are computed based on the statistical information extracted from the raw AA detections:

5 $$\begin{aligned} & w_{l} \left( k \right) = \left\{ {\begin{array}{*{20}l} {0,} \hfill & {0 \le k \le 70} \hfill \\ {\frac{{P_{meanAA} - P_{70} }}{meanAA - 70}\left( {k - 70} \right) + P_{70} ,} \hfill & {k \ge 70} \hfill \\ \end{array} } \right. \\ & w_{n} \left( k \right) = \left\{ {\begin{array}{*{20}l} {0,} \hfill & {0 \le k < 70} \hfill \\ {E\frac{{P_{meanAA} }}{{\sigma_{AA} }}{\text{exp}}\left( { - \frac{{\left( {k - meanAA} \right)^{2} }}{{2\sigma_{AA}^{2} }}} \right),} \hfill & {70 \le k \le meanAA} \hfill \\ {P_{meanAA} , } \hfill & { k \ge meanAA} \hfill \\ \end{array} } \right. \\ \end{aligned}$$

where $meanAA$ and $\sigma_{AA}$ represent the mean and standard deviation of the AA intervals, respectively. $P_{70}$ and $P_{meanAA}$ represent the weighting factor at time $t = 70$ ms and $t = meanAA$ ms.

The linear weight function $w_{l}$ is a ramp signal which starts from the value $P_{70}$ at time $t = 70$ ms and reaches the value $P_{meanAA}$ at $t = meanAA$ ms. The non-linear weight function $w_{n}$ is a truncated Gaussian distribution of mean $meanAA$ and standard deviation $\sigma_{AA}$, centered at $t = meanAA$ ms and enlarged by a factor $E$. For both weight functions, the null value during the first 70 ms ensures that no new activation is detected during this interval (physiological limit).

The post-processing is performed in two steps: false detections are removed first, and then, missed AAs are screened.

#### Correction of false atrial activations

As a first step and for each new AA detection, the two weight functions (Eq. 5) are computed for the preceding and succeeding interval around the current activation (LAT_0_). The amplitude of the previous and the next activation around the current activation is further multiplied by the weight functions. For the Rel-En algorithm, a detection is considered false and will be removed if the amplitude of the weighted activation is smaller than the threshold $TH_{P}$. For the AMM algorithm, the weighted signal is reprocessed by the detection algorithm and an AA detected on the original signal is considered false if it is no longer present on the weighted signal.

**Fig. 4:**
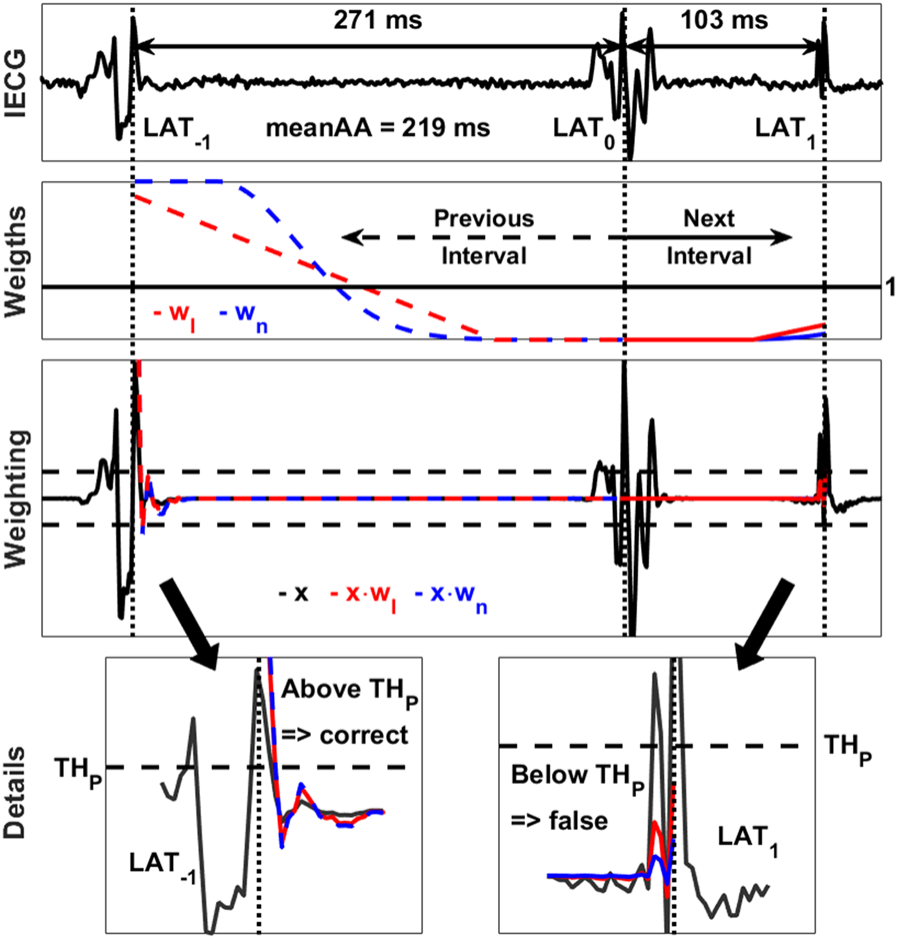
Correction of false AAs for the Rel-En algorithm. Three activations are detected on this time window (dotted vertical lines). The first two are true detections, the third one is an artifact. The top panel shows the IEGM. The weight functions computed around the processed activation LAT_0_ for the next and previous intervals are presented on the second panel. The linear and non-linear functions are displayed in red and blue, respectively. The effect of these weights on the output signal (in black) is shown on the third panel (red and blue for the linear and non-linear functions, respectively). Due to the shorter time interval (103 ms) between LAT_0_ and LAT_1_ compared to the mean value (219 ms), the amplitude at LAT_1_ is decreased by the weight functions and falls below the detection threshold. It is therefore considered as a false detection and removed from the AA sequence. The amplitude at LAT_−1_ is not reduced due to the large interval (271 ms) and this activation is kept as a correct detection. The effect on the activations LAT_−1_ and LAT_1_ is shown in detail on the last panel. AA, atrial activation; IEGM, intracardiac electrogram; LAT, local activation time; Rel-En, relative energy algorithm

An example of an artifact falsely detected as an AA with the Rel-En algorithm, and adequately removed using the weight functions is shown in Fig. 4. The weight functions (Eq. 5) are computed for the preceding interval $\left[ {LAT_{ - 1} , LAT_{0} } \right]$ and succeeding interval $\left[ {LAT_{0} ,LAT_{1} } \right]$ around the activation detected at time $LAT_{0}$. A value of these functions greater than 1 will result in an amplification of the signal, while a value smaller than 1 will result in a dampening of the signal. As shown in Fig. 4, a shorter time interval between $LAT_{0}$ and $LAT_{1}$ compared to the mean value estimated on all raw AA-intervals will result in a decrease in the amplitude by the weight functions at $LAT_{1}$ which will fall below the detection threshold $TH_{P}$. It will therefore be considered as a false detection and will be removed from the AA detections sequence.

#### Correction of missed atrial activations

As a second step, the interval between two consecutive activations at times $LAT_{i}$ and $LAT_{i + 1}$ is scrutinized to identify possible missed AA. The amplitude of the signal between the two activations is multiplied by the weight functions computed for the interval $\left[ {LAT_{i} , LAT_{i + 1} } \right]$:

6 $$\begin{aligned} x_{weighted} \left( k \right) & = W_{l|n} \left( k \right) \cdot x\left( {LAT_{i} + k} \right), \\ W_{l|n} \left( k \right) & = w_{l|n} \left( k \right) \cdot TR(w_{l|n} \left( k \right)), \\ \end{aligned}$$

where $x$ is the signal between the two activations, $w_{l|n}$ denotes the linear and non-linear weight function, respectively, and $TR(w_{l|n} \left( k \right))$ represents the time-reversed version of $w_{l|n} \left( k \right)$. For the Rel-En algorithm, an activation is considered as missed at the time $LAT_{i} + k$ if the value of $x_{weighted} \left( k \right)$ is greater than the threshold $TH_{P}$. For the AMM algorithm, the weighted signal is reprocessed by the detection algorithm to spot a possible missed activation.

**Fig. 5:**
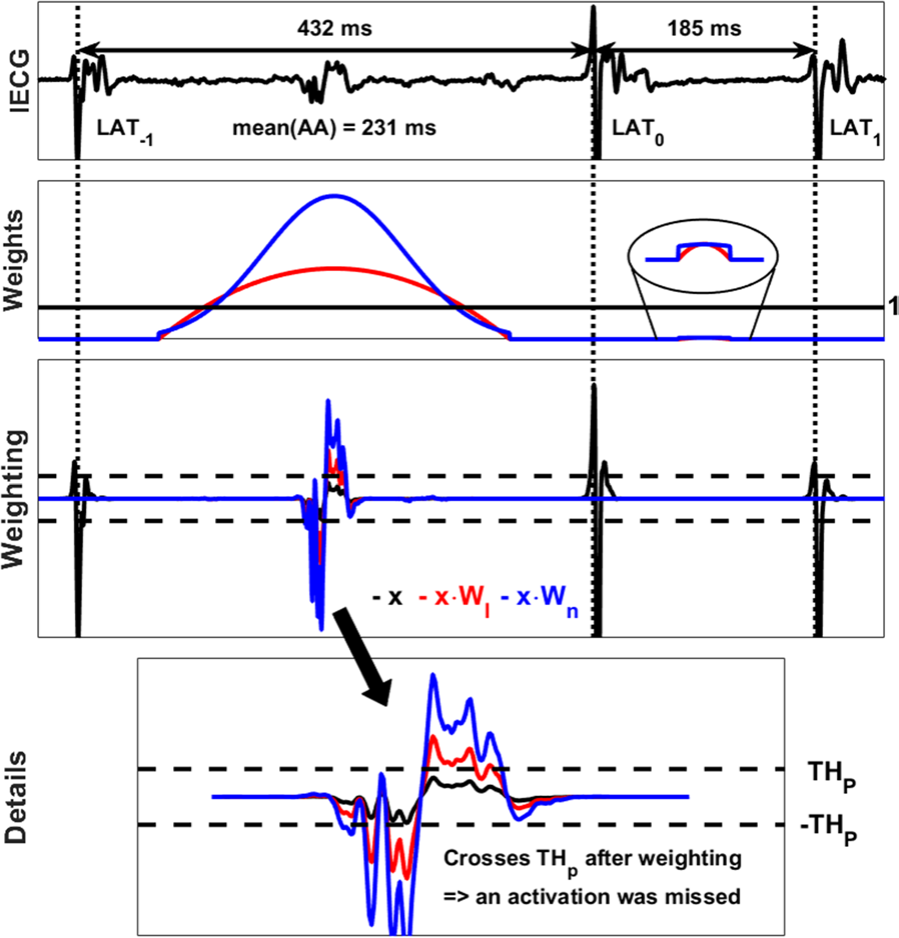
Correction of missed AAs for the Rel-En algorithm. Three activations are detected on this time window (dotted vertical lines). The top panel shows the IEGM. Second panel: combined weight functions $W_{l|n}$. (linear in red, non-linear in blue) Third panel: effect on the output signal (in black) of the linear (red) and non-linear (blue) weights for each interval. The first interval is long (432 ms) compared to the mean value (231 ms) of the AA intervals. The central part of the interval is then amplified and the weighted amplitude is above the detection threshold (horizontal dashed lines), revealing a missed activation. The second interval is short (185 ms), the signal is not amplified and no missed activation is detected. The last panel displays the missed activation in detail. AA, atrial activation; Rel-En, relative energy algorithm

An example of a detection missed by the Rel-En algorithm and corrected using the weight functions is shown in Fig. 5. After a first pass by the Rel-En algorithm, the signal is multiplied by the combined linear and non-linear weight functions. A value of the combined weight functions above 1 will result in amplification of the signal, while a value below 1 will result in dampening of the signal. As shown in Fig. 5, the first interval [LAT_−1_, LAT_0_] is longer than the mean value of the AA intervals (231 ms). This results in an amplification of the central part of the interval. The weighted version may hence cross the detection threshold, unmasking a missed activation. The newly detected activation will then be added to the AA sequence.

#### Comparison with existing algorithms

Our method was compared with two published algorithms on the validation cohort. Firstly, we applied the Cycle Length Iteration algorithm proposed by Ng et al. [19]. This method searches the peaks iteratively in the EGMs based on their amplitude. An ad-hoc criterion is proposed to determine the end of the detection. A post-processing step based on the AA intervals allows reducing the number of missed detections. Secondly, we used the Non Linear Energy Operator algorithm proposed by Nguyen et al. [18]. This method uses Teager operator to compute the energy of the signal. An adaptive threshold is applied on this energy signal to delimit the active zones of the signal corresponding to the AAs.

### Annotation correction

The presence of multiple peaks or fractionated signals on the IEGM may induce fluctuations in the timings of the AA detection. The temporal analysis of the AAs sequence is affected by these artificial variations. In order to reduce this effect, we propose a beat-by-beat correction of the AA annotation based on the following steps:

- Extract a portion of the signal around the AA
- Compute the envelope of the absolute value of the signal
- Reject low amplitude tails of the activation with a cutoff on the envelope
- Define the corrected annotation as the barycenter of the power of the remaining signal

The corrected annotation is computed on the squared signal, rather than on the signal itself, to enhance the influence of high amplitude peaks.

Since the exact times of the annotations are unknown, we use the variance of the AAs sequence to evaluate the result of the correction. Indeed, if we assume that the fluctuations of the annotations are independent of the AA, we have:

7 $$\begin{aligned} LAT\left( i \right) & = LAT_{0} \left( i \right) + e_{t} \left( i \right) + e\left( i \right), \\ var\left( {LAT} \right) & = var\left( {LAT_{0} } \right) + var\left( {e_{t} } \right) + var\left( e \right), \\ \end{aligned}$$

where $LAT\left( \cdot \right)$ is the sequence of AA times originally detected with the Rel-En or AMM algorithm, $LAT_{0} \left( \cdot \right)$ is the sequence of the true AA times, $e_{t} \left( \cdot \right)$ the sequence of the errors due to imprecisions in the estimation of the AA times and $e\left( \cdot \right)$ the sequence regrouping any other errors. Since the variance is positive, a reduction in the variance of the LAT sequence, $var\left( {LAT} \right)$, after the correction process corresponds to a reduction in the variance of $e_{t}$ sequence, i.e. the error due to the estimation of AA times. Due to the inherent non-stationarity of AF, the variance of the AA intervals is expected to be large. Our method corrects the annotations on a beat-to-beat basis, without considering the other activations. Therefore, with the hypothesis that the imprecisions of the detections are uncorrelated to the true variations of the AA sequences, the measured decrease in variance in the AA sequence indicates a reduction of this imprecision.

## Results

### Raw detection

#### Raw detection tuning

**Fig. 6:**
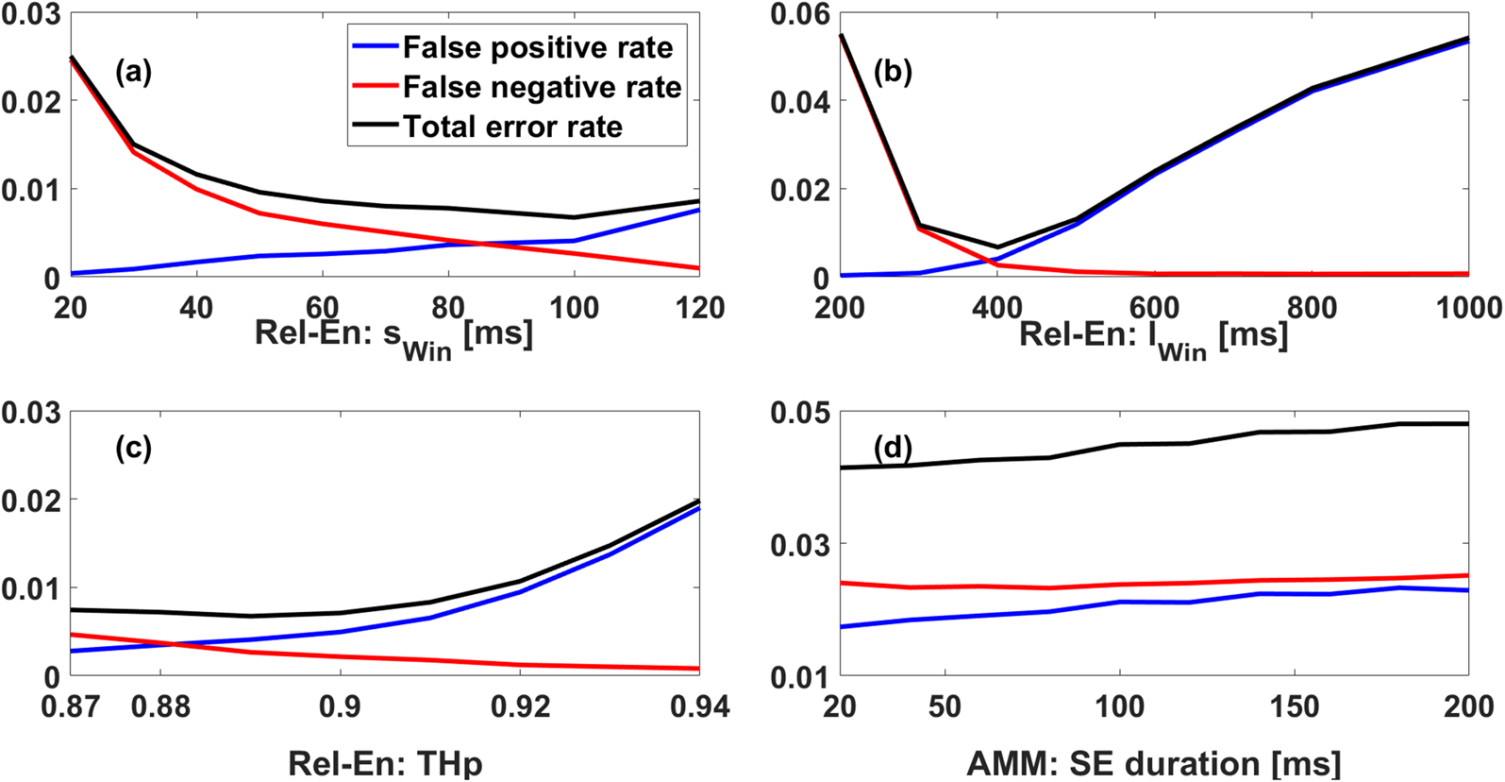
Detection error rates with respect to the parameters of the relative energy algorithm (**a**–**c**), and the adaptive mathematical morphology algorithm (**d**). The blue curve shows the false positive rate, the red curve shows the false negative rate. The total error rate is displayed in black

The Rel-En and AMM algorithms were optimized on the training cohort. The performances of the algorithms were assessed using the false negative/positive rates (number of missed/false detections divided by the number of activations) and the total detection error rate (the sum of false positive and negative rates). The impact of the parameters of the two algorithms on the detection error rates is displayed in Fig. 6.

*Relative-energy algorithm.* For the Rel-En algorithm, we simultaneously examined (grid search) the small and long window durations and the percentile value P. The small window $s_{win}$ was tested from 20 to 120 ms, the long window $l_{win}$ was tested from 100 to 1000 ms and the percentile value $P$ was tested from the 6th to 13th percentiles. The best parameters were defined as the ones yielding the fewest detection errors (i.e. false negative and false positive) on our training cohort.

Figure 6a–c show that the best result was obtained using $s_{win} = 100\;{\text{ms}}$, $l_{win} = 400\;{\text{ms}}$ and $P = 11th$ percentile. As shown in Fig. 6b the long window parameter $l_{win}$ has the largest influence on the total error rate in the vicinity of the optimal value for the Rel-En algorithm.

*Adaptive mathematical morphology algorithm.* Figure 6d shows the effect of the duration of the synthetized SE on the detection error rate. The length of SE was tested from 10 to 200 ms and the optimal duration giving the fewest detection errors was 20 ms.

#### Raw detection: comparison of the algorithms

Using the optimal parameters derived from the training cohort, we applied both algorithms on our validation cohort and computed the detection errors. The best detection was achieved using the Rel-En algorithm, yielding a false negative rate of 0.76% and a false positive rate of only 0.12%. The total error rate was 0.88%. The AMM algorithm yielded a poorer detection, achieving a false negative rate of 2.65% and a false positive rate of 0.84%. The total error rate was 3.49%. Results are summarized in Table 2.

**Table 2:** Performances of the Rel-En and AMM algorithms on the training and validation cohorts. The Rel-En algorithm performed better in the two cohorts both in terms of false negative and false positive rate

	Rel-En	AMM
**Training cohort**		
False negative	0.41%	1.74%
False positive	0.27%	2.40%
**Total error rate**	**0.67%**	**4.14%**
**Validation cohort**		
False negative	0.76%	2.65%
False positive	0.12%	0.84%
**Total error rate**	**0.88%**	**3.49%**

The bold typo is used to highlight the final error, the other errors are partial errors

### Reduction in over- and undersensing

After raw detection, post processing of AAs sequences was performed in order to reduce the detection errors. Parameters defining the weight functions were optimized on the training cohort for the results provided by both detection algorithms.

For the linear weight, the value at 70 ms ($P_{70}$) was tested from 0 to 0.4 and the value at the mean value of the AA sequence ($P_{meanAA}$) was tested from 1 to 3. For the nonlinear weight, the max weight value ($P_{meanAA}$) was tested from 1 to 3 and the enlargement parameter *E* was tested from 1 to 3.

#### Rel-En algorithm

For the Rel-En algorithm, the combinations of parameters providing the largest reduction in the detection errors were $P_{70} = 0$ and $P_{meanAA} = 2.1$ for the linear weight, and $E = 1.25$ and $P_{meanAA} = 3$ for the non-linear weight.

Table 3 summarizes the results achieved on the validation cohort using optimal parameters for both linear and non-linear weight functions. The linear weight provided a reduction of almost 50% in the false detection rate and 40% in the missed detection rate. The non-linear function yielded an even better improvement with an almost 70% reduction in both detection rates. The final total error rate using the non-linear weight fell to only 0.28%.

**Table 3:** Reduction in over- and undersensing. Detection optimization for the Rel-En and AMM algorithms using both linear and non-linear weight functions. The values in brackets refer to the relative variations in error rates with the optimization

	False negative (% change with optimization)	False positive (% change with optimization)	Total error (% change with optimization)
Raw Rel-En	0.76%	0.12%	0.88%
Linear	0.40% (− 47.4%)	0.07% (− 37.9%)	0.47% (− 46.1%)
Non-linear	0.24% (− 68.4%)	0.04% (− 70.6%)	0.28% (− 68.7%)
Raw AMM	2.65%	0.84%	3.49%
Linear	2.14% (− 19.2%)	1.17% (+ 39.3%)	3.31% (− 5.2%)
Non-linear	2.25% (− 15.1%)	1.08% (+ 28.6%)	3.33% (− 4.6%)

#### AMM algorithm

For the AMM algorithm, the combinations of parameters providing the largest reduction in the detection errors were $P_{70} = 0.4$ and $P_{meanAA} = 2.2$ for the linear weight, and $E = 1.25$ and $P_{meanAA} = 3$ for the non-linear weight.

Results achieved on the validation cohort using optimal parameters for both linear and non-linear weight shapes are summarized in Table 3. Both linear and non-linear weight functions increased the false detection rate while decreasing the missed detection rate of the algorithm. This resulted in a decreased total error rate in both cases. The linear weight performed slightly better than the non-linear weight. The reduction in total error rate obtained by the linear weight for the AMM was 5.2% compared to the raw algorithm.

### Comparison with existing algorithms

Two existing detection algorithms (CLI and NLEO) were applied on the validation cohort. The performances of the Rel-En algorithm after reduction of over- and under-sensing were compared to the those obtained with these two algorithms. The results are summarized in Table 4. The NLEO algorithm achieved a total error rate of 3.01% and the CLI algorithm achieved 3.63%. Both algorithms yielded higher rates of false positive and false negative detection compared to the Rel-En algorithm (*p* value < 0.05).

**Table 4:** Comparison results with existing algorithms. The Rel-En algorithm performed better, both in terms of false positive and false negative rates on the validation cohort

	CLI	NLEO	Rel-En
False negative	0.83%	1.77%	0.24%*
False positive	2.80%	1.24%	0.04%*
**Total error rate**	**3.63%**	**3.01%**	0.28%*

*p-value < 0.05 Rel-En versus CLI and Rel-En versus NLEO using Wilcoxon rank-sum test

The bold typo is used to highlight the final error, the other errors are partial errors

### Annotation correction

**Fig. 7:**
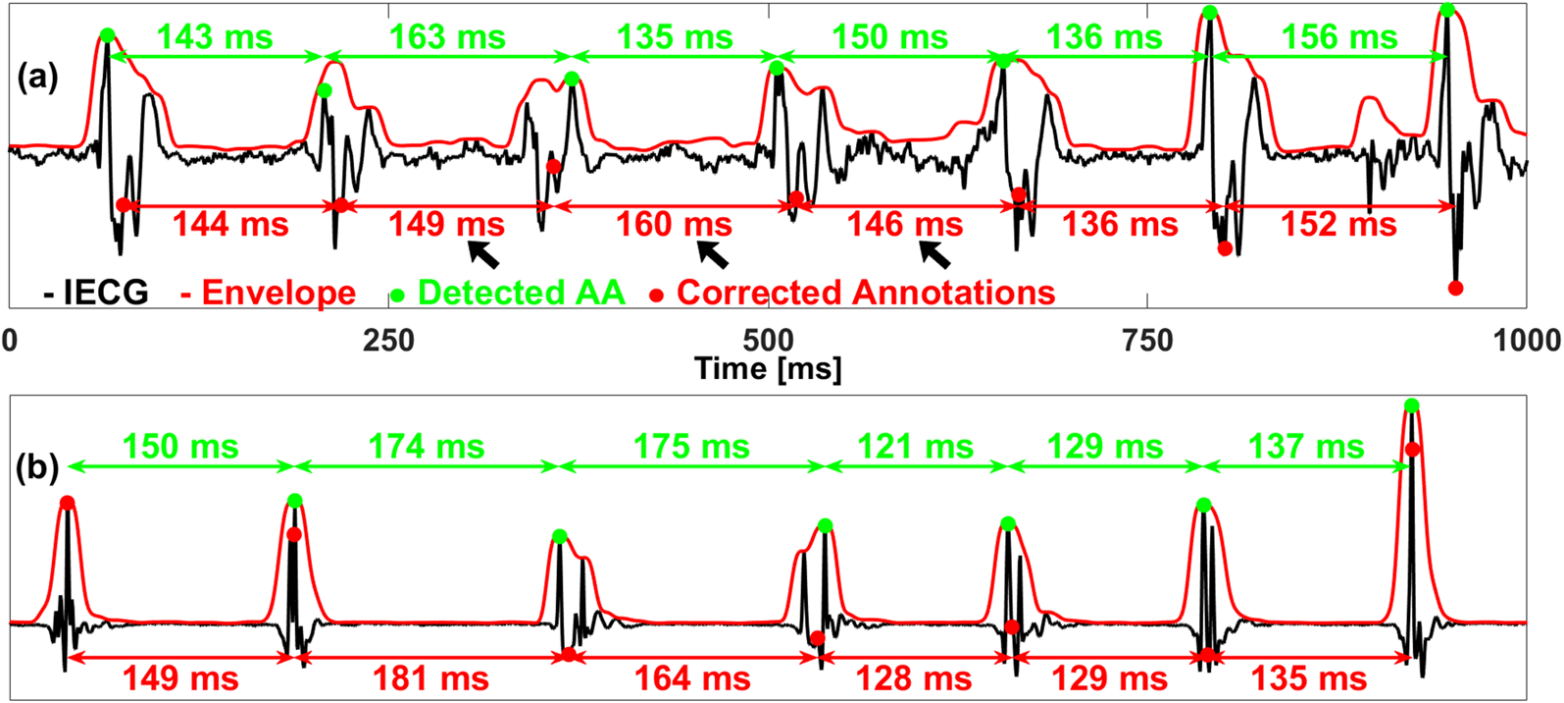
Examples of fractionated signals (**a**) and multiple peaks (**b**). The envelope of the signal is displayed in red. The detected atrial activations and the corresponding corrected annotations are represented by the green and red dots, respectively. Note on panel (**a**) that the peak annotation creates an artificial “long-short-long” sequence, while the correct sequence is actually a “short-long-short” one (black arrows)

In order to reduce the artificial variations in timings of the AA detection, a correction of these annotations was implemented on the optimized Rel-En algorithm. Figure 7 shows an example of the correction process in the case of fractionated signals (a) and in the presence of multiple peaks (b). The green dots represent the originally detected AAs, whereas the corrected annotations are displayed by red dots. The new annotations are less dependent on the shapes of the AAs, which lessens the artificially-induced variability in the AA-intervals sequence.

**Fig. 8:**
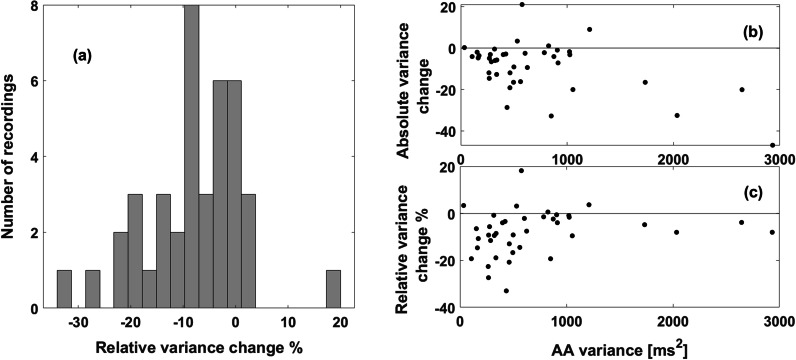
Variance changes resulting from the annotation corrections. The histogram of the relative variance changes is shown on panel (**a**). The absolute and relative changes are displayed with respect to the AA sequence variance on panels (**b**) and (**c**), respectively. Despite fairly large absolute variance changes, the relative changes in recordings with large AA variance were more limited. AA, atrial activation

On the validation cohort, a reduction of 5.4 ± 7.8% in the variance of the corrected AA-interval sequence was obtained compared to the original time series obtained by the Rel-En algorithm. In 17 records (42.5%), the reduction in the artificial variation was greater than 5%. In a single record, the correction process led to the opposite outcome, and resulted in an increase in variance greater than 5%. A histogram of the relative changes in variance is shown in Fig. 8a. The relative and absolute variance changes of each recording are displayed with respect to the original AAs variance of the recording on panels (b) and (c), respectively. Note that the recordings with a large variance display a small relative change even if the corresponding absolute change is large.

## Discussion

The present study proposes a novel iterative approach to automatically detect AAs and provide accurate CL estimations from bipolar IEGM recorded during persistent AF. The method is based on the following steps (illustrated in Fig. 1): (1) raw detection of AA complexes using a low-complexity algorithm recently developed by our group for the automatic extraction of events from biomedical signals; (2) correction for over- and under-sensing of AA detections using a novel processing technique consisting in a statistical-based weighting of the signal amplitude around each raw AA detection; (3) correction for the artificial variations in beat-to-beat CL estimations using a method based on the barycenter of the power of the AAs.

Our study shows that the proposed 3-step method is efficient for automatically detecting AAs and provides accurate beat-to-beat CL estimations on AF electrograms with complex activations and fractionation. Considering the marginal error rate achieved (0.28%), our method may be used for the automatic annotation of AAs on large database, allowing a huge time-saving compared to a manual verification. Furthermore, the low complexity of the signal processing techniques makes the proposed approach suitable for use in real-time/online settings. The Rel-En can be computed with a delay related to the length of the long-term window (400 ms) and the statistics used during the post-processing step could be updated in a buffer or computed along a sliding window.

The relative timings and morphologies of the activation patterns during AF are constantly changing which precludes the use of automated detection of individual AAs. Various methods have been previously proposed to automatically detect AAs from electrograms in AF. Threshold-based detection algorithms [10, 11], will invariably be prone to over- and undersensing due to the range of signal morphologies and amplitudes in AF. Ng et al. [19] introduced a new algorithm which uses a mean and median CL convergence criterion to detect atrial complexes. While the CL-based method may have advantages for IEGM with large beat-to-beat variations in CL, a threshold-based approach would be more appropriate for variable AA amplitudes. The Teager-Kaiser energy operator [12, 23], was proposed to detect abrupt changes in the signal amplitude and detect high-frequency events. This method is however sensitive to fractionation. More robust methods such as template matching [13] or wavelet transform have been proposed to deal with contaminated signals. However, the problem remains partially unsolved when dealing with signals with a large patient- and time-dependent spectrum of shapes, amplitudes and frequencies as is the case for intracardiac recordings of AF. Compared to these methods, our iterative approach has some advantages mainly due to the fact that the detection algorithms can be easily tailored for robust detection even for IEGMs with significant beat-to-beat variation both in CL and in AA morphology. Moreover, our detection algorithms require only a limited number of parameters to be optimized, and are easy to implement and computationally uncostly as demonstrated by Orlandic et al. [24]. In their study, a real-time ECG R-peak detection algorithm based on Rel-En produced comparable accuracy results with around one third less memory consumption compared to three state-of-the-art methods. The two window durations of the Rel-En algorithm can be intuitively selected based on the physiological constraints of the signals at hand. The Rel-En algorithm achieved the best performance for an optimal short-term and long-term window of 100 ms and 400 ms, respectively, which fits with the physiological constraints of the AA complexes in persistent AF. Moreover, due to the elementwise multiplication of the coefficient signal with the signal at hand, the algorithm is robust against spurious peaks as in the case of double-peak or fractionated activations. The AMM algorithm has been used in several previous studies and has already shown good results, especially in the detection of QRS complexes in ECG recordings. However, the variety of shapes in the intracardiac recordings alongside with the presence of impulsive and high frequency noise impair the performances of the algorithm. On the other hand, Rel-En is a recently developed algorithm aiming to enhance the events of interest in different types of physiological recordings, such as EEGs, PPG, etc. The high consistency shown by Rel-En in the automatic detection of activations against manually marked activations demonstrates the potential of our algorithm for the automatic detection of AAs during persistent AF. This has been confirmed by comparing the performances of our detection with two published algorithms. The Rel-En obtained superior results both in terms of false negative and false positive detection on the same set of recordings.

The Rel-En and AMM detection algorithms both showed significantly higher false negative than false positive rates. This is likely related to the high heterogeneity of AA morphology in persistent AF. Although the original algorithms yielded few false detections or missed activations, we further attempted to reduce the undersensing and oversensing. The proposed novel post-processing approach corrected almost all missed activations or false detections. The high correction efficiency may be explained by the fact that the approach is tailored to the processed IEGM. The two weight functions used to enhance the signal before and after each raw detected activation are computed using the mean value of the raw AA intervals. While using contextual information enables high correction efficiency, adequate recording length is needed for accurate estimation of mean CL. Recently, Ng et al. [19] also showed that the undersensing, oversensing and total error rate of their mean CL-based detection algorithm reached a stabilized level at an IEGM duration > 10 s.

Importantly, alongside a high sensitivity and specificity, the accurate estimation of the LATs is also a desired feature of the detection algorithms. The barycenter approach [25, 26], was proposed as a robust alternative to the classical peak or maximum slope annotation of the LAT. The main drawback of the barycenter approach is the estimation of the onset and offset of the activation for the barycenter determination. In the present study, we circumvented this barycenter-related issue by focusing on large amplitude portion of the activation, considering that the tails (or low amplitude parts) of the activation will only have a small influence in the LAT computation. This aspect was even reinforced by working with the squared envelope of the signal.

## Limitations

Our study may be limited by the size of the study population and by the fact that the detection approach was optimized and validated on a multi-center cohort consisting only of IEGMs recorded from the PVs. Since IEGM characteristics (including amplitude, fractionation, activation morphology) may vary depending on the structural and electric complexity of the underlying myocardium [27], our findings may not necessarily apply to highly fragmented or continuous signals recorded in the atria. Our database however includes electrograms of varied complex activation and morphological patterns, with heterogeneous signal amplitude and fractionation, but still with clearly present isoline. Furthermore, we also showed that variations in the algorithm parameters outside of the optimal values did not affect significantly the detection performance. Finally, a further limitation may relate to the fact that single expert annotation was used as ground truth.

## Conclusion

In the present study we propose a novel iterative approach to automatically detect atrial activations and provide accurate CL estimations from bipolar IEGMs recorded during persistent AF. By providing both a reliable detection and a robust temporal annotation of AAs, the added value of our proposed method resides in the fact that it addresses two main limitations encountered in the temporal analysis of AF activations. Considering the marginal error rate achieved and the low complexity of the signal processing technique, our proposed method may be used in real-time settings for the automatic annotation of AA on large databases.

## Data Availability

Data are available upon request.

## Abbreviations

AA: Atrial activation
AF: Atrial fibrillation
AMM: Adaptive mathematical morphology algorithm
LAT: Local activation time
CL: Cycle length
CLI: Cycle length iteration algorithm
IEGM: Intracardiac electrogram
NLEO: Non-linear energy operator algorithm
PV: Pulmonary vein
Rel-En: Relative-energy algorithm
